# Long-term outcome and predictors for recurrence after medical and interventional treatment of arrhythmias at the UniverSity Heart CenTer Hamburg (TRUST): design and patient profile snapshot of a prospective clinical cohort study

**DOI:** 10.1093/ehjopen/oeag002

**Published:** 2026-01-14

**Authors:** Julius Obergassel, Jan Leon Rieß, Sandro Jaeckle, Silke van Elferen, Moritz Nies, Niklas Schenker, Marc Daniel Lemoine, Alexander Welcker, Shinwan Kany, Laura Rottner, Djemail Ismaili, Laura Charlotte Sommerfeld, Johannes Petersen, Katharina Govorov, Lauritz Schoof, Olga Tsoy, Jan Baumbach, Simon Pecha, Tanja Zeller, Stefan Blankenberg, Larissa Fabritz, Bruno Reißmann, Feifan Ouyang, Andreas Rillig, Andreas Metzner, Paulus Kirchhof

**Affiliations:** Department of Cardiology, University Heart & Vascular Center Hamburg, University Medical Center Hamburg-Eppendorf, Martinistraße 42, 20246 Hamburg, Germany; German Center for Cardiovascular Research (DZHK), partner Site Hamburg/Kiel/Lübeck, Germany; Department of Cardiology, University Heart & Vascular Center Hamburg, University Medical Center Hamburg-Eppendorf, Martinistraße 42, 20246 Hamburg, Germany; German Center for Cardiovascular Research (DZHK), partner Site Hamburg/Kiel/Lübeck, Germany; Department of Cardiology, University Heart & Vascular Center Hamburg, University Medical Center Hamburg-Eppendorf, Martinistraße 42, 20246 Hamburg, Germany; Department of Cardiology, University Heart & Vascular Center Hamburg, University Medical Center Hamburg-Eppendorf, Martinistraße 42, 20246 Hamburg, Germany; German Center for Cardiovascular Research (DZHK), partner Site Hamburg/Kiel/Lübeck, Germany; Institute for Computational Systems Biology, Universität Hamburg, Albert-Einstein-Ring 8-10 22761 Hamburg, Germany; Department of Cardiology, University Heart & Vascular Center Hamburg, University Medical Center Hamburg-Eppendorf, Martinistraße 42, 20246 Hamburg, Germany; German Center for Cardiovascular Research (DZHK), partner Site Hamburg/Kiel/Lübeck, Germany; Department of Cardiology, University Heart & Vascular Center Hamburg, University Medical Center Hamburg-Eppendorf, Martinistraße 42, 20246 Hamburg, Germany; German Center for Cardiovascular Research (DZHK), partner Site Hamburg/Kiel/Lübeck, Germany; Department of Cardiology, University Heart & Vascular Center Hamburg, University Medical Center Hamburg-Eppendorf, Martinistraße 42, 20246 Hamburg, Germany; German Center for Cardiovascular Research (DZHK), partner Site Hamburg/Kiel/Lübeck, Germany; Department of Cardiology, University Heart & Vascular Center Hamburg, University Medical Center Hamburg-Eppendorf, Martinistraße 42, 20246 Hamburg, Germany; German Center for Cardiovascular Research (DZHK), partner Site Hamburg/Kiel/Lübeck, Germany; Department of Cardiology, University Heart & Vascular Center Hamburg, University Medical Center Hamburg-Eppendorf, Martinistraße 42, 20246 Hamburg, Germany; German Center for Cardiovascular Research (DZHK), partner Site Hamburg/Kiel/Lübeck, Germany; Department of Cardiology, University Hospital Frankfurt, Theodor-Stern-Kai 7, 60590 Frankfurt, Germany; Department of Cardiology, University Heart & Vascular Center Hamburg, University Medical Center Hamburg-Eppendorf, Martinistraße 42, 20246 Hamburg, Germany; German Center for Cardiovascular Research (DZHK), partner Site Hamburg/Kiel/Lübeck, Germany; Department of Cardiology, University Heart & Vascular Center Hamburg, University Medical Center Hamburg-Eppendorf, Martinistraße 42, 20246 Hamburg, Germany; German Center for Cardiovascular Research (DZHK), partner Site Hamburg/Kiel/Lübeck, Germany; German Center for Cardiovascular Research (DZHK), partner Site Hamburg/Kiel/Lübeck, Germany; Department of Cardiovascular Surgery, University Heart & Vascular Center Hamburg, University Medical Center Hamburg-Eppendorf, Martinistraße 42, 20246 Hamburg, Germany; Department of Cardiology, University Heart & Vascular Center Hamburg, University Medical Center Hamburg-Eppendorf, Martinistraße 42, 20246 Hamburg, Germany; Department of Cardiology, University Heart & Vascular Center Hamburg, University Medical Center Hamburg-Eppendorf, Martinistraße 42, 20246 Hamburg, Germany; Institute for Computational Systems Biology, Universität Hamburg, Albert-Einstein-Ring 8-10 22761 Hamburg, Germany; Institute for Computational Systems Biology, Universität Hamburg, Albert-Einstein-Ring 8-10 22761 Hamburg, Germany; Department of Mathematics and Computer Science, Computational Biomedicine Lab, University of Southern Denmark, Campusvej 55, 5230 Odense M, Denmark; Department of Cardiology, University Heart & Vascular Center Hamburg, University Medical Center Hamburg-Eppendorf, Martinistraße 42, 20246 Hamburg, Germany; German Center for Cardiovascular Research (DZHK), partner Site Hamburg/Kiel/Lübeck, Germany; German Center for Cardiovascular Research (DZHK), partner Site Hamburg/Kiel/Lübeck, Germany; Institute for Cardiogenetics, Institute for Genetics, University Hospital Schleswig-Holstein, Campus Lübeck, Building 67, BMF, Ratzeburger Allee 160, 23562 Lübeck, Germany; Department of Cardiology, University Heart & Vascular Center Hamburg, University Medical Center Hamburg-Eppendorf, Martinistraße 42, 20246 Hamburg, Germany; German Center for Cardiovascular Research (DZHK), partner Site Hamburg/Kiel/Lübeck, Germany; Department of Cardiology, University Heart & Vascular Center Hamburg, University Medical Center Hamburg-Eppendorf, Martinistraße 42, 20246 Hamburg, Germany; German Center for Cardiovascular Research (DZHK), partner Site Hamburg/Kiel/Lübeck, Germany; Institute of Cardiovascular Sciences, University of Birmingham, Edgbaston, Birmingham B15 2TT, UK; Department of Cardiology, University Heart & Vascular Center Hamburg, University Medical Center Hamburg-Eppendorf, Martinistraße 42, 20246 Hamburg, Germany; German Center for Cardiovascular Research (DZHK), partner Site Hamburg/Kiel/Lübeck, Germany; Department of Cardiology, University Heart & Vascular Center Hamburg, University Medical Center Hamburg-Eppendorf, Martinistraße 42, 20246 Hamburg, Germany; Department of Cardiology, University Heart & Vascular Center Hamburg, University Medical Center Hamburg-Eppendorf, Martinistraße 42, 20246 Hamburg, Germany; German Center for Cardiovascular Research (DZHK), partner Site Hamburg/Kiel/Lübeck, Germany; Department of Cardiology, University Heart & Vascular Center Hamburg, University Medical Center Hamburg-Eppendorf, Martinistraße 42, 20246 Hamburg, Germany; Department of Cardiology, University Heart & Vascular Center Hamburg, University Medical Center Hamburg-Eppendorf, Martinistraße 42, 20246 Hamburg, Germany; German Center for Cardiovascular Research (DZHK), partner Site Hamburg/Kiel/Lübeck, Germany; Institute of Cardiovascular Sciences, University of Birmingham, Edgbaston, Birmingham B15 2TT, UK

**Keywords:** cardiac arrhythmias, Atrial fibrillation, Prospective cohort study, Trial design, Biobank, Wearables

## Abstract

**Aims:**

Optimal outcomes for patients with cardiac arrhythmias can be achieved through multimodal therapy including lifestyle support, medication, and interventions. Planning of these therapy concepts requires a detailed understanding of phenotypes, responses to therapies, and outcomes.

**Objective:**

The prospective Long-term Outcome and Predictors for Recurrence after Medical and Interventional Treatment of Arrhythmias at the University Heart Center Hamburg (TRUST) combines deep phenotypic, procedural, and follow-up information in a contemporary cohort of patients with cardiac arrhythmias.

**Methods and results:**

TRUST is an investigator-initiated, prospective, cohort study enrolling consecutive patients at the UHZ Hamburg. Comprehensive baseline work-up, imaging, and biobanking at baseline is combined with follow-up using a combination of in-person visits, online questionnaires, and remote rhythm-monitoring. Enrolment started in March 2021 and is ongoing. This paper describes the design and the clinical characteristics of the first 1500 enrolled patients with verified baseline datasets (562 women (37%), median age 64 (IQR 55, 74) years). Overall, 1077/1500 patients (71%) were seen for atrial fibrillation, 161/1500 (11%) for ventricular tachycardia and premature ventricular complexes, 239/1500 (16%) for supraventricular tachycardia, and 23/1500 (2%) patients for other reasons. Ablations, rhythm surgery, or invasive procedures were performed in 1363/1500 (91%) within 1 month after inclusion.

**Conclusion:**

This snapshot of the first 1500 patients enrolled in TRUST illustrates current characteristics and comorbidity burden in patients undergoing arrhythmia treatment. This rich data set will provide information on treatment patterns, detailed phenotypes, and follow-up, offering insights into the natural progression and treatment responses of arrhythmias in routine care.

## Graphical Abstract

**Graphical Abstract:**
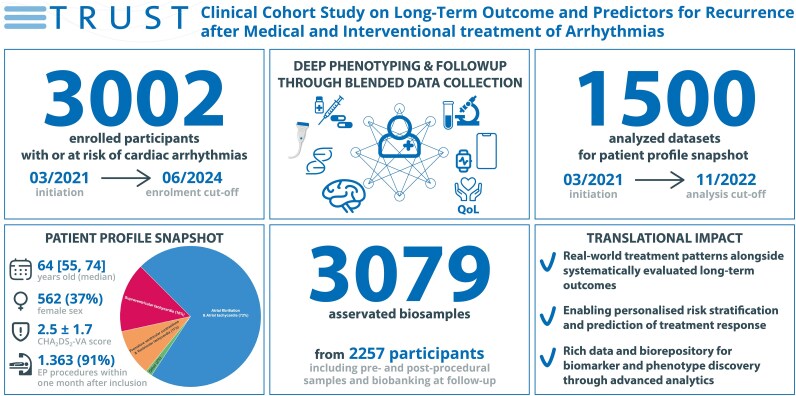

What’s New?

- This paper introduces TRUST, a newly established, contemporary cohort for patients with or at risk of cardiac arrhythmias, featuring deep phenotyping, systematic biobanking, and long-term follow-up across a broad arrhythmia spectrum.
- Blended data collection and follow-up systems integrate fully into clinical workflows, increasing efficiency and enabling sustained, high-quality data acquisition in routine care settings.
- Over two-thirds of patients in a contemporary arrhythmia department are referred for atrial fibrillation (71%), with significant proportions for ventricular (11%) and supraventricular tachycardias (16%).
- The patient profile snapshot reveals increasing median age (64 (IQR 55, 74) years) and comorbidity burden, alongside lower antiarrhythmic drug use at admission, compared to most atrial fibrillation ablation trials, which reflects the advancing evidence on early atrial fibrillation ablation in patients with a high comorbidity burden compared to traditional patient selection approaches for atrial fibrillation ablation.
- A high rate of guideline-recommended medical therapies, including anticoagulation and heart failure medication, is observed at admission for TRUST's baseline visit, reflecting their broad uptake in real-world clinical settings.
- High patient willingness to participate in extensive, digitally supported research and biobanking creates a rich data and biorepository as a foundation supporting advanced analytics, translational hypothesis, and real-world evidence generation, biomarker discovery, and development of personalized arrhythmia management strategies.

## Introduction

Cardiac arrhythmias are among the common chronic cardiovascular disorders with important associated morbidity, mortality, and healthcare-related costs,^1,2^ including atrial fibrillation (AF), ventricular tachycardia (VT), premature ventricular complexes (PVC), and supraventricular tachycardia (SVT). Most patients with arrhythmia require integrated, comprehensive, ‘holistic’ care. In atrial fibrillation, this combines therapy of concomitant cardiovascular condition, oral anticoagulation, early rhythm control,^3,4^ including AF ablation,^5,6^ rate control (add Kotecha RATE-AF JAMA2020).^7–9^ Improved safety and efficacy of ablation of ventricular tachycardias and PVC ablation led to its broader adoption, combined with treatment of underlying cardiomyopathies and cardiovascular conditions.^10^ In parallel, insights into the genetic causes of arrhythmias and efforts to integrate quantitative traits for the description of subphenotypes hold promise for precision therapy^11^ of arrhythmias and challenge long-standing concepts in arrhythmia management, such as AF classification and indications for oral anticoagulation.^12^ Innovations in ablation technologies^13,14^ and progress in remote monitoring using medical systems and consumer electronics^15–17^ improve access to early diagnosis and to interventional therapy. This enhances the need for data-driven personalized therapy selection. The ‘Long-Term Outcome and Predictors for Recurrence after Medical and Interventional treatment of Arrhythmias at the UniverSity Heart CenTer Hamburg’ prospective clinical cohort study (TRUST) integrates detailed patient phenotypes, procedural information and follow-up in consecutive participants with, or at risk for, cardiac arrhythmias treated at the University Heart and Vascular Center Hamburg into a detailed phenotypic picture of contemporary patients with arrhythmias seen in a tertiary care center. Here, we report the design of TRUST and a snapshot of baseline data of the first 1500 patients enrolled until 11/2022.

## Methods

### Study setup, patient population, and ethical considerations

**Figure 1:**
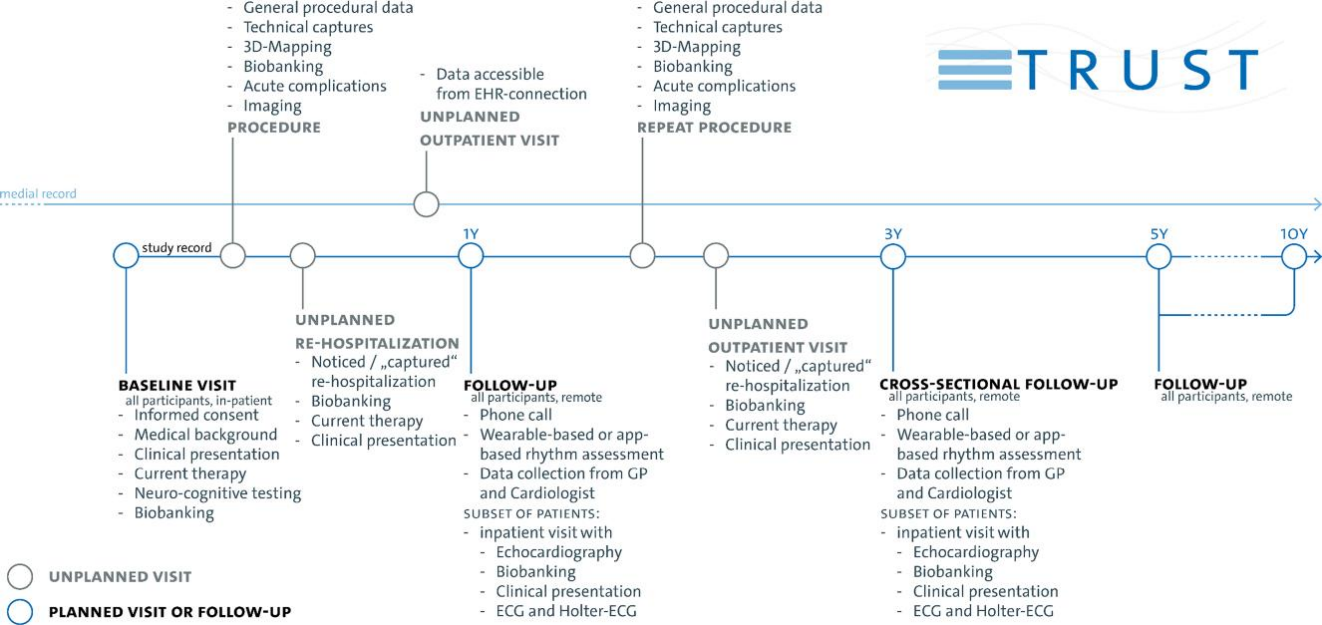
Design and timeline of TRUST including all investigations planned. Follow-ups are being conducted systematically as defined by the study protocol after one year, five years and ten years. A cross-sectional follow-up is being conducted in a similar fashion but with an increased number of participants returning to the center for in-house investigations around three years after initiation of TRUST. Additionally, planned and unplanned re-hospitalizations or outpatients are captured via a deep integration of the data platform with the clinical’s digital information system.

TRUST is an investigator-initiated, single-center, prospective clinical cohort study acquiring long-term follow-up information on participants with or at risk for cardiac arrhythmias. The study is registered at ClinicalTrials.gov under NCT05521451. An overview of the study protocol is visualized in *Figure 1*. Study procedures are described in *Table 3*.

The study enrolls consecutive consenting adult patients seen for arrhythmia therapy at the University Heart and Vascular Center Hamburg (UHZ), a large tertiary medical center in Northern Germany. Pre-defined exclusion criteria are insufficient knowledge of the German language or other factors precluding appropriate informed consent and follow-up. TRUST is approved by the ethics committee of the Ärztekammer Hamburg, Germany, in 2020 (processing no# 2020-10066-BO-ff) with two protocol amendments regarding smartphone- and wearable-based remote follow-up. TRUST adheres to the Declaration of Helsinki, Good Clinical Practice guidelines, and local legislation.

### Recruitment, informed consent, inclusion, pseudonymization

Patients are invited to participate by clinical staff and trained medical students through different channels: (1) the arrhythmia clinic’s call-in service distributes TRUST brochures, consent forms and contact details, (2) patients are invited to participate in TRUST during outpatient or inpatient visits for arrhythmia treatment at the UHZ, and (3) treating physicians at UHZ discuss participation in TRUST with their patients. Written informed consent is obtained from all patients before enrolment.

The University Heart and Vascular Center Hamburg (UHZ) runs multiple clinical cohorts, among them those including patients suffering from acute heart failure (HF), chronic HF, presenting with suspected myocardial infarction, undergoing interventions for valvular heart disease, and others. Within the past 2 years, a common core variable set plus a shared pseudonymization and data infrastructure for all clinical cohorts was established, allowing for cross-linking of participants and analyses across all clinical cohorts.

### Sub-cohort tailor study pathways

All patients are followed up for core information, including cardiovascular events, hospitalizations, recurrent arrhythmias, and quality of life. Depending on their chief-complaint upon initial presentation, additional disease-specific information is captured for atrial fibrillation or atrial tachycardia (AF/AT), patients with AF undergoing open-heart surgery and concomitant AF ablation (Surgery), supraventricular tachycardia (SVT), ventricular tachycardia or premature ventricular complexes (VT/PVC), or inherited cardiac conditions (ICC).

### Pre-specified research objectives

TRUST is not designed to address a single predefined hypothesis but instead enables both hypothesis-driven and exploratory analyses within a large, deeply phenotyped cohort, as well as adaptations over time to adjust to new scientific advances. However, several objectives and analyses were pre-specified in the study protocol before data collection for the cross-sectional follow-up and are summarized in *Table 1*. These objectives guided the design of the data collection instruments in TRUST’s follow-up. Additionally, outcome definitions were pre-specified as outlined in *Table 2*.

**Table 1:** Pre-specified objectives and planned analyses of the TRUST clinical cohort study

Domain	Prespecified research objective	Subgroup	Key Outcome(s)
PVI	Comparing procedural success, long-term rhythm control, and safety of different PVI modalities	AF patients undergoing first PVI	AF/AT recurrence Cardiovascular events Re-hospitalizations Procedural complications and safety Repeat procedure occurrence and characteristics
	Identification of clinical, imaging, electrophysiological and autonomic predictors of long-term success in rhythm-control therapy		
	Quantify acute and longer-term autonomic modulation induced by different PVI modalities and explore their effect on AF recurrence		
HF & AF ablation	Impact of HF-directed medical therapy at discharge on HF- and rhythm-outcomes after ablation	Patients with AF and concomitant HF (including HFpEF) undergoing AF ablation	Adherence to administered medication Recurrence of AF/AT Cardiovascular events Re-hospitalizations Quality-of-life (QoL) Changes in functional assessments and biomarker profiles
	Quantification of left-ventricular ejection fraction response to rhythm-control therapy in suspected arrhythmia-induced cardiomyopathy	Patients with AF undergoing AF ablation with a reduced left-ventricular ejection fraction at baseline	Change in left-ventricular ejection fraction at follow-up HF medication use at follow-up Changes in functional assessments and biomarker profiles Cardiovascular events Re-hospitalizations
VT ablation	Compare procedural and long-term outcomes of VT ablation in ischemic vs. non-ischemic cardiomyopathy	Patients undergoing VT ablation	Sustained VT recurrence ICD therapies Cardiovascular events Re-hospitalizations
Obstructive sleep apnea and AF	Influence of obstructive sleep apnea (and CPAP therapy) on outcomes after left-atrial ablation	Patients with AF/AT and concomitant OSA undergoing AF ablation	AF/AT recurrence Procedural complications and safety Cardiovascular events Antiarrhythmic drug use AF burden
Disease progression & incident events	Identification of predictors of new-onset arrhythmias, new-onset heart failure, and progression of arrhythmia burden over time	Entire cohort stratified by arrhythmia sub cohort	Incident AF/AT, VT, HF, decline of kidney function Changes in arrhythmia burden Cardiovascular events Re-hospitalizations
Patient-reported outcomes & neurocognitive function	Assess determinants and longitudinal change in quality of life, symptom burden, mental health and neurocognitive status; correlate AF burden to symptom burden; identification of patient subgroups with highest symptomatic benefit; feasibility of experience sampling in AF research and reassessment of QoL instruments	Entire cohort stratified by arrhythmia sub cohort (with completed questionnaires and follow-ups)	Change in QoL instruments (AFEQT, EQ-5D-5L) Change in PHQ-9 Change in neurocognitive performance (MoCA/MiniMoCA) Symptom burden
Endophenotyping and therapy individualization through novel biomarkers, AF burden and integration of autonomic function	Explore associations between circulating biomolecules and further digital, genetic, molecular biomarkers with clinical phenotypes of outcomes, arrhythmia recurrence and disease progression	Entire cohort stratified by arrhythmia sub cohort (with available biomarkers)	Arrhythmia recurrence Cardiovascular events Re-hospitalizations Change in biomarker profiles Disease progression; AF burden
	Quantify AF burden over time; correlate burden with outcomes; evaluate impact of individualized therapy based on burden/phenotype; assess feasibility and prognostic value of wearable- and patient-operated device monitoring	Sub-cohort enrolled in wearable/digital health monitoring (existing cohort plus planned extension)	AF burden metrics AF/AT recurrence Cardiovascular events QoL Changes in antiarrhythmic and HF therapy
	Assess whether baseline autonomic state before PVI identifies patients who derive greater benefit from thermal compared to non-thermal ablation	AF patients undergoing first PVI	Arrhythmia recurrence Change in biomarker profiles Chage in heart rate variability

Research objectives are displayed categorized by domain. The planned analysis subgroup, as well as related key outcomes are displayed.

AF, Atrial fibrillation; AT, Atrial tachycardia; AFEQT, Atrial Fibrillation Effect on QualiTy-of-life questionnaire; CPAP, Continuous positive airway pressure (ventilation); EQ-5D-5L, European Quality of Life 5-Dimension 5-Level survey; HF, Heart failure; HFrEF, Heart failure with reduced ejection fraction; HFpEF, Heart failure with preserved ejection fraction; MoCA, Montreal Cognitive Assessment; OSA, Obstructive sleep apnea; PHQ-9, Patient Health Questionnaire-9 item depression scale; PROM, Patient-reported outcome measures; PVI, Pulmonary vein isolation; VT, Ventricular tachycardia.

**Table 2:** Key outcome measures assessed through TRUST’s long-term follow-up

Outcome		Pre-specified definition/means of assessment
Main outcome	A composite of	
	cardiovascular death,	Patient-reported; GP-/HCP-reported Center’s electronic patient record Resident registration data query
	ischemic stroke,	Patient-reported; GP-/HCP-reported Center’s electronic patient record
	acute coronary syndrome, or	Patient-reported; GP-/HCP-reported Center’s electronic patient record
	hospitalization for heart failure	Patient-reported; GP-/HCP-reported Center’s electronic patient record
Secondary outcome measures	Arrhythmia burden at baseline and follow-up, assessed through ECG monitoring, wearables and digital applications*	Center’s Holter-ECGs at baseline and follow-up; Center’s device interrogations at baseline and follow-up GP-/HCP-collected Holter-ECGs at baseline and follow-up; GP-/HCP-collected device interrogations at baseline and follow-up Wearable-devices administered to wearable sub-cohort in TRUST Smartphone-interrogations with data from patient-operated and -owned wearables
	Quality-of-life measures (AFEQT, EQ-5D-5L, PHQ-9)	Patient-reported through self-administered online or written questionnaires and complemented in follow-up telephone calls
	Patient-reported outcome measures (PROMs)	Patient-reported through self-administered online or written questionnaires and complemented in follow-up telephone calls
	Time to recurrent enrollment arrhythmia	Patient-reported; GP-/HCP-reported; Center’s electronic patient record including ECGs, Holter-ECGs Wearable-devices administered to wearable sub-cohort in TRUST
	Time to diagnosis of new arrhythmias	Patient-reported; GP-/HCP-reported; Center’s electronic patient record including ECGs, Holter-ECGs Wearable-devices administered to wearable sub-cohort in TRUST
	Time to hospitalization for heart failure	Patient-reported; GP-/HCP-reported; Center’s electronic patient
	Time to incident heart failure	Patient-reported; GP-/HCP-reported; Center’s electronic patient
	Worsening or improvement of left-ventricular function during follow-up*	Center’s electronic patient record with study-embedded (sub cohorts) or routinely performed follow-up echocardiography
	Worsening or improvement of kidney function during follow-up	Patient-reported; GP-/HCP-reported Center’s electronic patient record and embedded laboratory data
	Time to death and cause of death	Patient-reported; GP-/HCP-reported Center’s electronic patient record Resident registration data query
	Time to complications of rhythm control therapy	Patient-reported; GP-/HCP-reported Center’s electronic patient record
	Health care utilization during follow-up	Patient-reported; GP-/HCP-reported Center’s electronic patient record
	Decline of cognitive function (MoCA)	Telephone-based MoCA version
	Adherence to administered medication and digital health applications	Medication: Patient-reported Digital health applications: Utilization data

The additional information in brackets indicates the assessment tools used for the respective outcome measure. The asterisk (*) indicates that these outcome measures will only be available to a subset of patients, e.g., those who receive onsite follow-up investigations beyond remote follow-ups, or those taking part in TRUST’s wearable sub-cohort.

AFEQT, Atrial Fibrillation Effect on Quality-of-Life questionnaire; ECG, electrocardiogram; EQ-5D-5L, EuroQol 5-Dimension 5-Level questionnaire; MoCA, Montreal Cognitive Assessment; PHQ-9, Patient Health Questionnaire-9; PROM, Patient-reported outcome measures.

### Outcomes

The *main outcome* of TRUST is the time to a composite of cardiovascular death, ischemic stroke, acute coronary syndrome, or hospitalization for HF. The *co-main outcome* is the time to new onset of other cardiac arrhythmias during follow-up. Further secondary outcome measures and their means of assessment are outlined in *Table 2*.

### Baseline assessment

Baseline assessment includes physical examination, symptom assessment, vital signs and anthropometry, medical history and current therapy (medication and device therapy), neurocognitive status (through Montreal Cognitive Assessment, MoCA), a 12-lead electrocardiogram (ECG) and transthoracic echocardiography. Participants answer additional demographic, symptom, and quality-of-life-related questionnaires. Blood results from clinically indicated analyses are integrated into the registry. Additional blood samples for research analyses are separated into serum and plasma from all consenting patients. Genomic DNA is extracted from the buffy coat. These biomaterials are stored at −80°C in a central biorepository.

### Follow-up assessments

**Figure 2:**
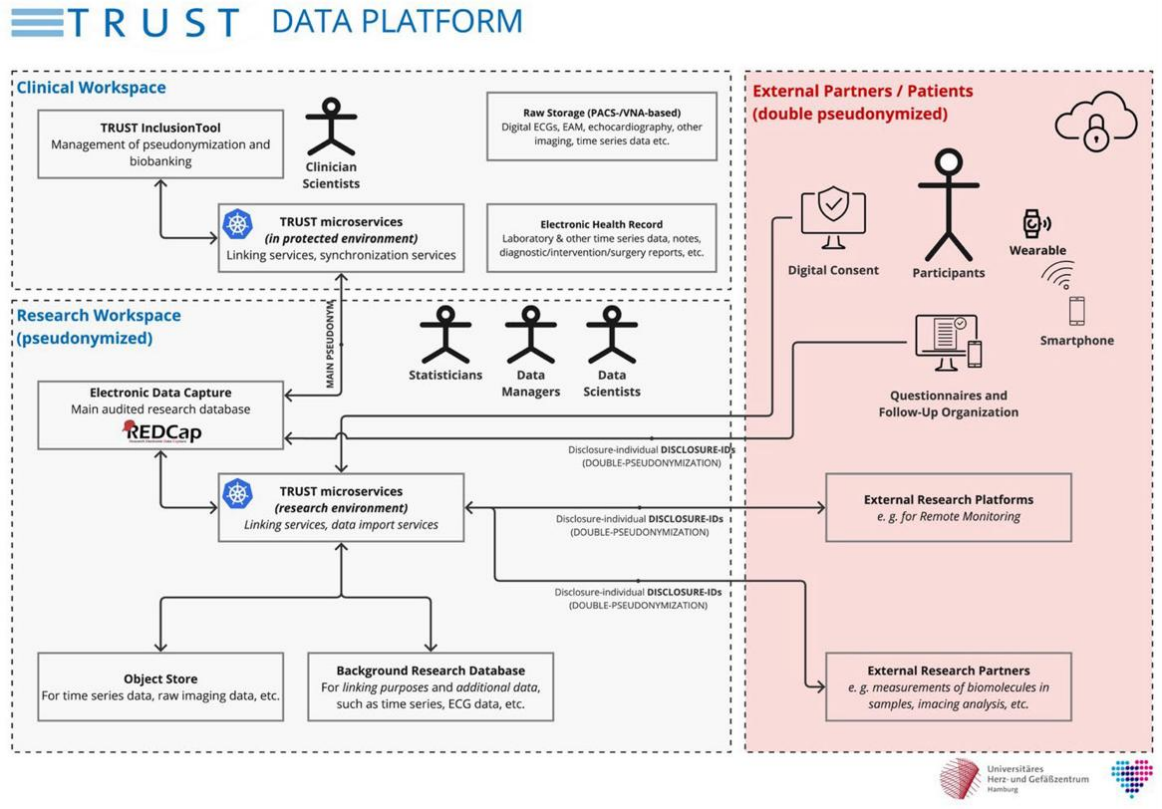
The TRUST data platform allows for linking of original clinical and pseudonymized research data, collaboration with external partners, as well as direct interaction with participants via their smartphones and through web-based platforms. The clinical workspace air-gapped, as well as network-separated from the research workspace, which has limited internet access and therefore allows for import of research-data from collaboration partners. Pseudonyms are generated and stored safely in the air-gapped clinical workspace (TRUST InclusionTool). They are automatically pushed into the REDCap-based data capture platform in the research workspace via TRUST microservices. Additional raw clinical data, such as imaging or electrocardiograms, can be exported and pseudonymized semi-automatically. Remote consent to TRUST sub-studies, e. g. wearable-based monitoring, is handled via a two-factor-authentication based system. External research platforms and internal or external collaboration partners are involved only via project-individual unique disclosure identifiers (DisclosureIDs) which ensures that the main pseudonyms are never disclosed to third parties, not even the participant.

Participants are invited to regular follow-ups, with the TRUST data platform capturing routine clinical data throughout the study period. It is planned to recall all patients for a follow-up visit 1 year, 5 years, and 10 years after inclusion. A comprehensive cross-sectional follow-up through all participants was conducted 3.5 years after initiation of the project in Q3/2024 and Q2/2025 (*Figure 1*, *Table 3*). Sub-studies embedded within TRUST involve additional follow-up protocols, including repeated blood sampling and more detailed follow-up investigation. Outcomes are captured through a combination of scheduled telephone interviews, patient questionnaires (completed online or in written form), correspondence with general practitioners, and remote monitoring via smartphone-based fingertip-PPG rhythm monitoring, guided by telephone support where needed. Remote monitoring includes remote consent and automatic assignment of a second pseudonym (double pseudonymization), managed through custom-built software with web interfaces hosted at the University Medical Center Hamburg-Eppendorf (*Figure 2*). To minimize loss to follow-up, patients receive advance postal notification of upcoming telephone interviews together with access to online questionnaires, followed by repeated, usually five, call attempts. Resident registration data are queried to verify vital status in non-reachable patients and events, and the cause of death was ascertained from records received from general practitioners or involved specialists. Remaining alive non-responders are contacted postally with a shortened print-version questionnaire.

**Table 3:** Planned and unplanned follow-ups within TRUST, with investigations being conducted

	1Y (post inclusion)	3Y (post initiation)	5Y (post inclusion)	10Y (post inclusion)
**Remote Follow-Ups** − Telephone interview − Inquiry at general practitioners − Querying national death registry − One-month smartphone-based RRM	All participants		All participants	All participants
**In-House Follow-Ups** + Transthoracic echocardiography + 18-hours Holter-ECG + Device interrogation + Biobanking	Subgroups		Subgroups	Subgroups
**Cross-sectional remote follow-up** − Telephone interview − Inquiry at general practitioners − Querying national death registry − One-month smartphone-based RRM		All participants		
**Cross-sectional in-house follow-up** + Transthoracic echocardiography + 18-hours Holter-ECG + Device interrogation + Biobanking		Subgroups G1: Index PVI G2: AF + EF < 50% at inclusion		
**(Un)planned visits at UHZ** − Interview − Transthoracic echocardiography − Biobanking (with consent)	All patients; triggered by visit			

While the 1-, 5-, and 10-year follow-ups are related to each individual patient’s inclusion date, a cross-sectional follow-up in all patients was conducted. Besides, certain subgroups of patients enrolled in the TRUST registry that were part of clinical studies conducted under the umbrella of TRUST received additional in-house routine follow-up. These groups include patients undergoing first-time pulmonary vein isolation (PVI) for atrial fibrillation (AF) (index PVI), patients with suspected arrhythmia-induced cardiomyopathy at inclusion, and a subgroup of patients who were monitored with smartphone-coupled wearables following index PVI.

Y, year; ECG, electrocardiogram; G{N}, Group {N}; RRM, Remote rhythm monitoring utilizing a validated CE-marked smartphone application which captures and analyzes fingertip photoplethysmography recordings.

### Specific assessment protocols

Please refer to Text Supplement  *S1* in the Supplementary material online, Material for more detailed protocols and references for specific study-related assessments.

### Integrative data platform

All data collected for TRUST are combined and stored in a secure research infrastructure at the University Medical Center Hamburg-Eppendorf in pseudonymized form. Data protection policies, as well as technical and organizational measures, were reviewed by the responsible data protection officer at University Medical Center Hamburg-Eppendorf. Whenever possible, pseudonymization and transfer are automated or semi-automated, a) to improve quality via reduction of manual transfer-related errors and b) to enhance privacy by limiting the extent of involvement of study personnel.

Manually collected variables and results of study-related non-routine investigations are captured by trained study personnel using REDCap as a central electronic data capture platform.^18^ Other study data that cannot be sensibly recorded in REDCap, such as large tabular sets of laboratory data, imaging data, time-series data, or data from electroanatomical mapping studies, are stored for further analysis in TRUST’s research database as well as an S3-based object store linked to the patient pseudonyms. For feature extraction from and classification of unstructured data and certain clinical texts, TRUST’s research infrastructure employs natural-language-processing techniques, (small) language models, as well as deep learning (ref. Text Supplement  *S2*).

TRUST's data platform also facilitates remote consent for sub-studies and enables the distribution of online questionnaires during follow-up. Remote consent employs two-factor authentication, using a credential known only to the participant and a second factor delivered by mail. The platform integrates third-party tools, such as web services of the fingertip- and wearable-PPG providers using automatic double pseudonymization, data retrieval, and their integration with the study database.

REDCap’s MyCap extension is used during remote follow-up for regular, continuous collection of patient-reported outcome measures and symptoms.^17^ All external partners, external platforms, and communication with participants are handled via specific unique identifiers, different from the main pseudonyms. This ensures that the primary pseudonyms are never disclosed to third parties, not even to the participant.

## Data quality, validation and planned statistical analysis

The statistical analysis of TRUST is conducted by a team of trained statisticians, bioinformaticians, and experienced data scientists, in addition to machine learning architects at the University Medical Center Hamburg-Eppendorf and selected collaboration partners. All data stored in the data platform is monitored continuously and at milestones via pre-configured rule-based algorithms and statistical plausibility and integrity checks. Flagged or missing values is periodically reviewed against source documentation and corrected in the main database with audit-trail documentation.

All analyses of the TRUST cohort and its sub-cohorts will be defined in pre-specified analysis plans, starting with the analysis of the main outcome that includes the follow-up data collected in 2024 and 2025 and is expected in 2026. Double-checked missing data will be handled according to prespecified analysis plans using complete-case analyses, explicit missingness categories where appropriate, or imputation strategies. Multiple imputation can be applied when methodologically justified, whereas variables with non-imputable missingness will be excluded from specific models. Sensitivity analyses will be performed whenever feasible to assess the robustness of findings.

Outcomes will be evaluated using Cox proportional hazard models, incorporating clinical covariates and biomolecule concentrations. The first primary endpoint will be analyzed stratified by the main study cohort. Cumulative incidence curves will be presented using the Aalen-Johansen estimator to account for death as a competing event. Outcomes, including death, will be displayed using Kaplan-Meier curves. Sub-analyses of TRUST will be conducted in accordance with the specific clinical questions and datasets under investigation, including access to imaging and electroanatomical mapping data, analyses of digital ECGs, and analyses of the biosamples. All microservices to handle data processing within TRUST’s data platform are built in Python 3^19^ and deployed via Docker.^20^

## Statistical analysis of the current cohort profile

This paper reports baseline characteristics of the first 1500 patients. These were validated in a comprehensive data review and monitoring process in Q1 and Q2 2025. Descriptive statistics were used to describe the characteristics of the sub-cohorts. Cohort differences were compared univariately by Chi-squared tests for categorical variables and common parametric and non-parametric tests for the comparison of the central tendency. All statistical analyses in this report were performed using Python 3.12, SciPy, scikit-learn, and matplotlib for plotting.

## Results

### Patients

**Figure 3:**
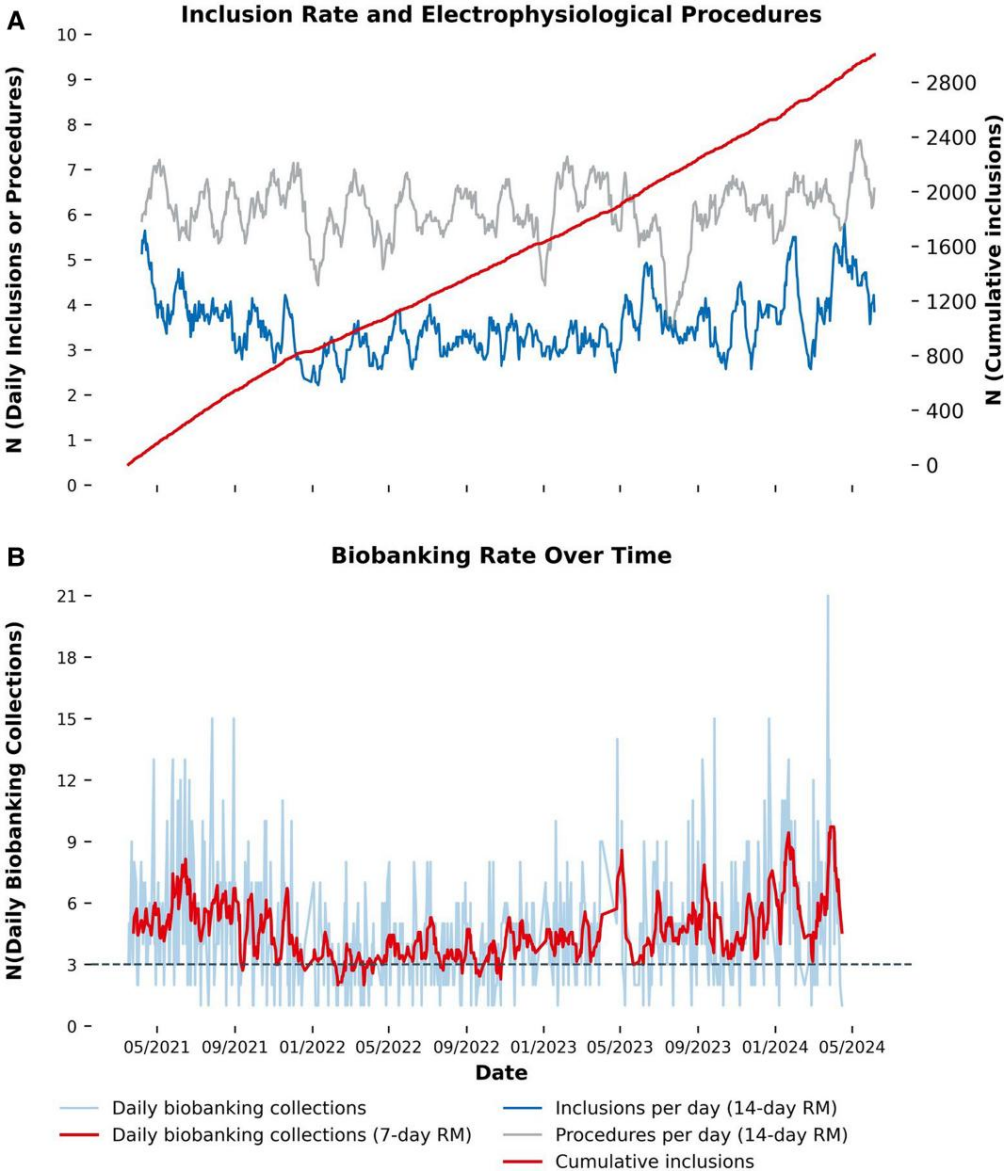
Inclusion and biobanking overview. Inclusion rate related to the number of performed electrophysiological procedures since initiation of the TRUST study on 17 March 2021 (2A), biobanking rate since initiation of TRUST (2B). The inclusion line is displayed as a rolling mean over 14 days of the daily inclusion rate (blue line), a line of cumulative inclusions (red line) as well as a rolling mean over 14 days of daily performed electrophysiological procedures. The daily biobanking rate is evenly displayed along with a red line indicating a rolling mean over 7 days.

The first patient was enrolled in TRUST on 17 March 2021. Inclusion of new patients was paused on 17 June 2024 after enrolment of 3000 patients. Inclusion rate was stable over time (*Figure 3A*) at a median rate of 19 participants per week. To assess potential sources of selection bias, inclusion rates and reasons for non-participation were evaluated in a predefined recruitment window from January 1st until 15 March 2023. Herein, 340 patients were approached for inclusion after excluding those who did not show up to their elective appointments. Of these, 37/340 (11%) were already enrolled at prior visit, while 175/340 (51%) were newly enrolled, consented, and completed the baseline assessment. Non-participation (128/340; 38%) was occasionally due to patient refusal in 21/340 (6%), including 3/21 (14%) due to language barriers, but more often due to workflow-related constraints during pre-procedural evaluations 107/340 (31%).

### Biosamples

By June 2024, 3079 biosamples from 2257 participants have been collected and stored at −80°C in a standardized, central biorepository (*Figure 3B*). Biobanking at baseline is currently available from 2146 (72%) participants. Pre- and post-procedural blood sampling is available from 239 procedures in 239 participants before and after ablation. Opportunistic follow-up samples (n = 277) have been collected from 255 participants at future hospitalizations or study-related follow-up visits, with a median biobanking follow-up time of 11 [IQR: 6;13] months after inclusion.

### Baseline characteristics of the first 1500 patients

**Figure 4:**
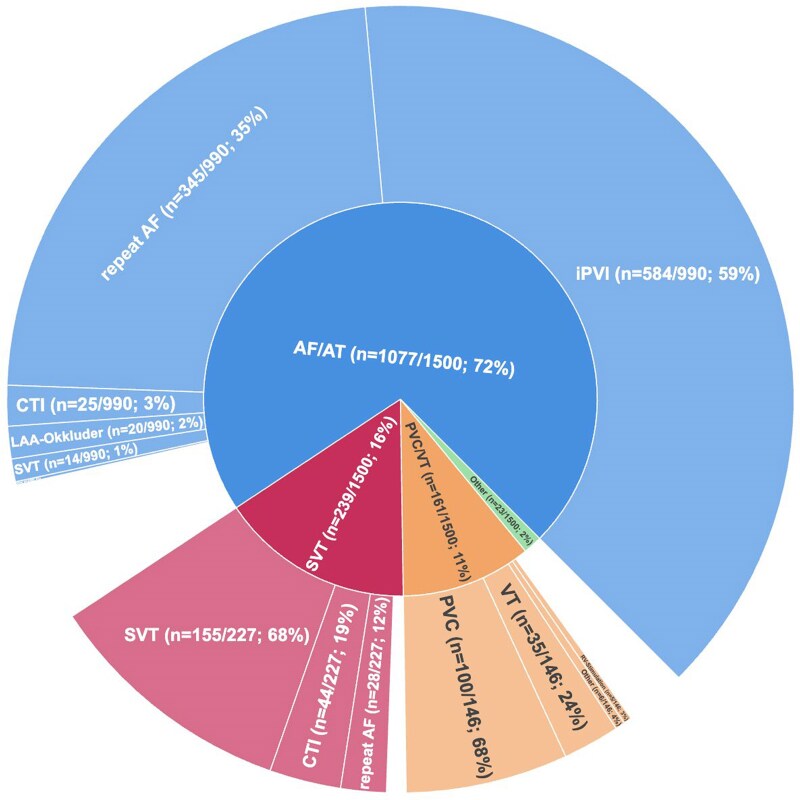
Procedures performed within 1 month after inclusion. Patients enrolled into the registry were assigned to chief-complaint cohorts (Atrial fibrillation or AF-related atrial tachycardia (AF/AT, blue), non-AF-related supraventricular tachycardia (SVT, red), Premature ventricular complexes or ventricular tachycardia (PVC/VT, orange), Inherited cardiac conditions and Heart surgery—subsumed in one category (‘Other’, green)). The sunburst plot shows cohort assignments upon inclusion in the inner circle and interventional procedures performed within 1 month after inclusion in the outer circle. Procedure categories with less than 20 patients were subsumed (‘Other’). Blank spaces in the outer circle indicate the share of patients without an interventional procedure performed within 1 month after inclusion. AF, Atrial fibrillation; AT, Atrial tachycardia; CTI, cavotricuspid isthmus; PVC, premature ventricular complexes; (i)PVI, (index) pulmonary vein isolation; SVT, supraventricular tachycardia; VT, ventricular tachycardia.

Validated and clean data from the first 1500 patients were compared by presenting arrhythmia. Most patients (1077/1500 (72%)) presented with AF or atrial tachycardias (AT). SVT or non-AF-related AT was the presenting arrhythmia in 239 (16%) patients, and VT or PVC was the main arrhythmia in 161/1500 (11%). Inherited cardiac conditions (9 patients (1%)) and AF-related cardiac surgery (14 patients 1%)) were rarer hospitalization causes at enrollment. Median age upon inclusion was 64 years [IQR: 55, 74], and 562 (37%) were women. Distribution of comorbidities varied between subgroups. The highest age and comorbidity burden was observed in the heart surgery and the AF/AT sub-cohorts (*Table 4*). A history of coronary artery disease, coronary intervention, or myocardial infarction was most common in patients with VT/PVC. Coronary artery disease was present in 51/161 (32%) of VT/PVC participants and 286/1077 (27%) of the AT/AF participants (p_AF/AT-vs-VT/PVC_ < 0.001). Prior coronary intervention was reported in 52/161 (33%) of VT/PVC participants 190/1077 (18%) of the AF/AT participants (p_AF/AT-vs-VT/PVC_ < 0.001), while history of myocardial infarction was present in 24/161 (15%) of VT/PVC participants and 84/1077 (8%) of AF/AT participants (p_AF/AT-vs-VT/PVC_ < 0.001, *Table 4*). Most patients (91%) underwent ablation or electrophysiological testing within 1 month after enrolment (*Figure 4*, *Table 5*).

**Table 4:** Baseline characteristics, comorbidities and echocardiography measurements of the first 1500 patients included in TRUST between March, 2021, and March, 2022

	Overall	AF and AT	VT and PVC	Non-AF SVTs	ICC	Surgery
N participants (%)	1500 (100)	1077 (72)	161 (11)	239 (16)	9 (1)	14 (1)
Age, N (%)	64 [55,74]	67 [58,75]	58 [47,70]	52 [35,65]	33 [27,59]	66 [60,77]
Female sex, N (%)	562 (37)	385 (36)	56 (34)	111 (46)	8 (88)	2 (14)
Body mass index (kg/m^2^)	27 [24,30]	27 [24,31]	26 [23,30]	26 [23,30]	24 [21,27]	26 [23,28]
CHA_2_DS_2_-VA Score, median and mean	2 [1,4] 3 (2)	3 [2,4] 2 (2)	2 [1,3] 2 (2)	1 [0,2] 1 (1)	0 [0,0] 0 (0)	4 [2,5] 4 (2)
CHA_2_DS_2_-VA Score ≥ 2, N (%)	1022 (68)	845 (78)	80 (50)	85 (36)		12 (86)
Heart rhythm						
Atrial fibrillation, N (%)	1121 (75)	1077 (100)	34 (21)	0 (0)	0 (0)	10 (71)
Paroxysmal atrial fibrillation, N (%)	446 (30)	424 (40)	16 (10)	0 (0)		6 (43)
Months since first diagnosis, N (%)	12 [4,41]	12 [4,41]	24 [14,51]			6 [6,6]
Atrial flutter, N (%)	296 (20)	235 (22)	1 (0.6)	59 (25)		1 (7)
Prior left atrial tachycardia, N (%)	111 (7)	107 (7)	3 (1.9)	1 (0.4)		
Prior sustained ventricular tachycardia, N (%)	86 (6)	20 (1.8)	58 (36)	5 (2.1)	1 (11)	2 (14)
Prior catheter ablation, N (%)	517 (34)	435 (29)	43 (26)	38 (16)		1 (7)
Echocardiography						
Left ventricular ejection fraction, N (%)						
≥50%, N (%)	1086 (72)	786 (73)	88 (54)	196 (82)	9 (100)	10 (71)
40–49%, N (%)	153 (10)	118 (11)	18 (11)	15 (6)		2 (14)
30–39%, N (%)	109 (7.0)	86 (8.0)	19 (12)	4 (1.7)		
<30%, N (%)	77 (5.0)	51 (4.7)	19 (12)	6 (2.5)		1 (7)
Diastolic septal wall width, mm	11.0 (3.2)	11.3 (2.8)	10.1 (1.8)	10.4 (4.8)	8.7(1.5)	11.8 (1.6)
Right ventricular pressure, systolic, mmHg	25 [20,32]	26 [22,33]	22 [18,27]	22 [18,29]	18 [18,18]	28 [21,45]
Left atrial volume, indexed, mL/m^2^	33 [24,42]	35 [27,44]	30 [23,39]	24 [19,29]	18 [17,22]	49 [42,58]
Present Heart Disease						
Heart failure, N (%)	894 (60)	725 (67)	90 (56)	67 (28)		12 (86)
HFpEF, N (%)	548 (37)	466 (43)	30 (19)	43 (18)		9 (64)
HFmrEF, N (%)	158 (11)	122 (11)	20 (12)	14 (6)		2 (14)
HFrEF, N (%)	188 (13)	137 (9)	40 (25)	10 (4.1)		1 (7)
Coronary artery disease, N (%)	369 (25)	286 (26)	51 (32)	23 (10)		9 (64)
Prior myocardial infarction, N (%)	117 (8)	84 (8)	24 (15)	9 (3.8)		
Valvular heart disease, N (%)	326 (22)	256 (24)	39 (24)	20 (8)		11 (79)
Medical History						
Arterial hypertension, N (%)	921 (61)	751 (70)	72 (44)	87 (36)	1 (11)	10 (71)
Diabetes mellitus, N (%)	168 (11)	125 (12)	18 (11)	20 (8)		5 (36)
Hypercholesterinemia, N (%)	560 (37)	449 (42)	61 (38)	41 (17)		9 (64)
Peripheral artery disease, N (%)	42 (2.8)	37 (3.4)	3 (1.9)	1 (0.4)		1 (7)
Pulmonary embolism, N (%)	43 (2.8)	33 (3.1)	2 (1.3)	8 (3.3)		
Chronic obstructive pulmonary disease, N (%)	55 (3.6)	47 (2.4)	5 (3.1)	2 (0.8)		1 (7)
Obstructive sleep apnea syndrome, N (%)	106 (7)	92 (9)	8 (5)	6 (2.5)		
Estimated glomerular filtration rate (CKD-EPI), mL/min/1.73 m^2^	72 (22)	71 (20)	79 (23)	92 (20)	93 (17)	53 (24)
Prior major bleeding (ISTH), N (%)	31 (2.0)	29 (2.7)	1 (0.6)	1 (0.4)		
Prior stroke or TIA, N (%)	112 (8)	93 (9)	10 (6)	7 (2.9)		2 (14)
Any malignant disease, N (%)	191 (13)	155 (14)	18 (11)	16 (7)		2 (14)
Rheumatological disease, N (%)	50 (3.3)	38 (3.5)	5 (3.1)	6 (2.5)		1 (7)
Hyperthyroidism, N (%)	59 (3.9)	47 (4.3)	8 (5)	4 (1.7)		
Hypothyroidism, N (%)	71 (4.7)	39 (3.6)	14 (9)	18 (8)		

Data is displayed stratified by the chief-complaint cohort assigned at inclusion into the TRUST registry.

AF, Atrial fibrillation; HF, Heart failure; HFmrEF, Heart failure with mildly reduced ejection fraction; HFpEF, Heart failure with preserved ejection fraction; HFrEF, Heart failure with reduced ejection fraction; ICC, Inherited cardiac condition; ISTH, International Society of Thrombosis and Hemostasis; SVT, Supraventricular tachycardia; VT, Ventricular tachycardia.

**Table 5:** Electrophysiological procedures performed within one month after inclusion in the atrial fibrillation (AF) or AF-related atrial tachycardia (AT) chief-complain cohort, the ventricular arrhythmias cohort (PVC/VT), and the non-AF-related supraventricular tachycardia cohort (SVT) in TRUST

	AF and AT	VT and PVC	Non-AF SVTs
**N patients in cohort (%)**	1077 (72)	161 (11)	239 (16)
**N patients with an interventional procedure performed within one month after inclusion**	990/1077 (92)	146/161 (91)	227/239 (95)
First pulmonary vein isolation	584/990 (59)		
Repeat pulmonary vein isolation Repeat atrial fibrillation ablation Focal or macro-reentry atrial tachycardia ablation (left and right)	345/990 (35)		28/227 (12)
Typical atrial flutter ablation	25/990 (3)		44/227 (19)
Ablation of other SVT	14/990 (1)		155/227 (68)
PVC ablation		100/146 (68)	
VT ablation		35/146 (24)	
Programmed stimulation without ablation		5/146 (3)	
Left atrial appendage occlusion	20/990 (2)		
Other procedures	2/990 (0)	6/146 (4)	
**No procedure performed within one month after inclusion**	87/1077 (8)	15/161 (9)	13/239 (5)

AF, Atrial fibrillation; AT, Atrial tachycardia; CTI, Cavotricuspid isthmus; LAA, Left atrial appendage; PVC, Premature ventricular complexes; SVT, Supraventricular tachycardia; VT, Ventricular tachycardia.

### Atrial fibrillation and atrial tachycardia

Of 1077/1500 (72%) patients enrolled for AF or AF-related AT, 644/1077 (60%) had non-paroxysmal patterns of AF. A history of left-, right- or bi-atrial focal or macro-reentrant AT in relation to AF diagnosis was present in 107/1077 (10%) patients. Median time from AF diagnosis to TRUST inclusion was 12 [4;41] months, 10 [2;19] months for patients with paroxysmal AF, 15 [5;47] months for participants with non-paroxysmal AF, and 15 [4;48] months for participants undergoing index PVI (irrespective of AF pattern). Most patients underwent ablation procedures within a month (990/1077 (92%)). Most procedures (n = 584/990, 59%) were first-time AF ablation, 345/990 (35%) were repeat AF ablation procedures (including repeat PVI, AT ablation in AF patients, AV-nodal ablation and combined procedures), 25/990 (3%) were ablations of the cavo-tricuspid isthmus for atrial flutter, 14/990 (1%) were ablation for other SVTs in AF patients, and 20 (2%) of patients underwent interventional left atrial appendage closure. More than three-quarters 738/945 (78%) of patients undergoing AF or repeat AF ablation (945/1077, 88%; *Table 5*) had a CHA_2_DS_2_-VA Score of 2 or higher. A diagnosis of heart failure was present in 725/1077 (72%) patients. Thereof, a reduced ejection fraction (HFrEF) was present in 137/725 (19%) and a mid-range ejection fraction (HFmrEF) in 122/725 (17%) of HF patients. Most HF patients (466/725; 64%) had a preserved ejection fraction (HFpEF). Cardiomyopathies were reported in 282/1077 (26%) patients, split into ischemic cardiomyopathy in 77/1077 (7%), dilatative cardiomyopathy in 60/1077 (6%), and hypertrophic cardiomyopathy in 32/1077 (3%).

Anticoagulation rate of AF patients that underwent first-time or repeat AF ablation (929/1077) was high at enrolment: 679/725 (94%) patients with an indication for long-term anticoagulation (CHA_2_DS_2_-VA ≥ 2) were on anticoagulation, and 800/929 (86%) in the overall cohort of patients undergoing AF or repeat AF ablation. OAC rate increased to 925/929 (100%) at discharge. Out of 925/929 patients receiving OAC at discharge, 885 (96%) were taking direct oral anticoagulants (DOAC). Further details of medical therapy at admission and changes at discharge are outlined in *Table 6*. Flecainide or propafenone were prescribed to 105/929 (11%) at admission and 84/929 (9%) at discharge, with 54/105 (51%) discontinued prescriptions and 33/824 (5%) newly initiated prescriptions. Amiodarone, dronedarone, or sotalol were prescribed to 109/929 (12%) at admission and 164/929 (18%) at discharge, with 38/109 (35%) discontinuations and 93/820 (11%) new initiations, predominantly of amiodarone. Betablockers were given to 753/929 (81%) patients at admission, discontinued in 26/753 (3%) patients and newly initiated in 27/176 (15%) patients.

**Table 6:** Admission and discharge medication of patients undergoing ablation for atrial fibrillation (AF) or ventricular tachycardia (VT) within one month after inclusion per sub-cohort in TRUST

	Baseline first-time or repeat AF ablation (n = 929)				Baseline VT ablation (n = 35)			
	Admission	Discharge	Discontinued	Initiated	Admission	Discharge	Discontinued	Initiated
RAASi	49% 455/929	51% 470/929	6% 27/455	9% 42/474	37% 13/35	26% 9/35	38% 5/13	5% 1/22
MRA	16% 145/929	20% 184/929	2% 3/145	5% 42/784	46% 16/35	46% 16/35	6% 1/16	5% 1/19
ARNI	9% 85/929	13% 121/929	1% 1/85	4% 37/844	37% 13/35	51% 18/35	8% 1/13	27% 6/22
SGLT2i	9% 83/929	15% 135/929	1% 1/83	6% 53/846	29% 10/35	51% 18/35	0% 0/10	32% 8/25
Betablocker	81% 753/929	81% 754/929	3% 26/753	15% 27/176	97% 34/35	94% 33/35	6% 2/34	100% 1/1
Flecainide or Propafenone (Class I AAD)	11% 105/929	9% 84/929	51% 54/105	4% 33/824	0% 0/35	0% 0/35	0% 0/0	0% 0/0
Amiodarone, dronedarone, sotalol (Claas III AAD)	12% 109/929	18% 164/929	35% 38/109	11% 93/820	40% 14/35	46% 16/35	14% 2/14	19% 4/21
OAC	86% 800/929	100% 925/929	0% 3/800	99% 128/129	29% 10/35	37% 13/35	0% 0/10%	12% 3/25

Patients enrolled in the respective chief-complaint cohort but not undergoing interventional treatment were excluded from this analysis. Discontinued prescriptions were calculated as the percentage of patients that had the respective prescription at admission but not at discharge. Initiations were calculated as the percentage of patients that did not receive the respective medication at admission but at discharge.

ARNI, Angiotensin-receptor neprilysin inhibitor; AF, Atrial fibrillation; MRA, Mineralocorticoid receptor antagonist; OAC, Oral anticoagulation; RAAS, Renin-angiotensin-aldosterone-system; SGLT2i, Sodium glucose transporter 2 inhibitor; VT, Ventricular tachycardia.

### Ventricular arrhythmias

Ventricular arrhythmias were the chief-complaint at enrollment in 161/1500 patients (11%). Of these, 35/161 (22%) underwent VT ablation within 1 month after inclusion. Most patients undergoing VT ablation were male (n = 30; 86%), and their median age was 59 years [55,70]. Heart failure was present in 21/35 (62%), often with a reduced ejection fraction below 40% (HFrEF, 17/33 patients (51%; *P* < 0.001 compared to patients with AF/AT). Most patients had ischemic heart disease (23/35; 66%) with established coronary artery disease, a prior coronary intervention, or a history of myocardial infarction (14/35; 41%). Dilated cardiomyopathy was present in 10/35 (29%), hypertrophic cardiomyopathy in 1/35 (3%), and 4/35 (12%) patients underwent ablation for VT without structural heart disease (idiopathic VT).

Amiodarone was already prescribed to 14/30 (46%) of patients with VT at enrolment and in 15/30 (50%) patients at discharge after VT ablation, with 2 discontinuations and 3 new initiations. Beta-blockers were given to 29/30 (97%) patients at enrolment and to 28/30 (94%) patients at discharge, with 2 discontinuations and one new prescription. High new initiation rates were noted for sacubitril/valsartan (27% new prescriptions in patients without a prescription at admission) and sodium-glucose-cotransporter-2 inhibitors (32%, *Table 6*).

Another 100/161 (61%) of patients were scheduled for PVC ablation within 1 month after inclusion. Of these, 8/100 (8%) did not receive ablation because PVCs could not be mapped. In the remaining 92 procedures, PVCs originated from the right or left ventricular outflow tract (including intramural origins) or left ventricular summit in 33/92 (36%) patients, from the aortic cusps in 13/92 (14%) patients, from papillary muscles in 9/92 (10%) patients, and from various other locations (including parahisian PVCs, inferobasal LV, and other) in 37/95 (40%) patients.

## Discussion

### TRUST—a large contemporary single-center arrhythmia cohort

TRUST achieved recruitment of 3000 participants in 3 years, in line with its aim to enroll consecutive patients with arrhythmias. The cohort is broadly representative of contemporary patients seen in similar centers, with 71% enrolled for AF or AT, 16% for SVT, and 11% for VT or PVC. Patient refusal to participate in TRUST was rare, even in temporary low-recruitment periods, whereas workflow-related constraints (107/340, 31%) accounted for most non-participation.

### Design aspects and digital architecture

High rates of biomaterial collection (72%) and procedural documentation underscore the feasibility of integrating routine care and advanced phenotyping for downstream research purposes through a blended data collection system, combining the work of study personnel with automated capture of routine data online, as well as patient-operated elements. This approach enables a broad capture of phenotypes, risk factors, symptoms, neurocognitive status and quality-of-life, relying on both patient-reported data and enabling structured assessments in routine care. Early inclusion metrics support TRUST's capacity to inform real-world arrhythmia outcomes and therapy pathways. First analyses of baseline data combined with detailed imaging phenotypes already enabled quantification of the presence of heart failure with preserved ejection fraction in patients with AF.^21^ TRUST’s digital architecture encompasses the use of wearables and further mobile health technology, as well as structured remote follow-ups which enables individualized, tailored follow-up pathways for distinct patient groups. Overall, TRUST’s protocolized, prospective, structured study-specific patient assessment is superior to retrospective derivation of these data from electronic health records. Via ongoing integration of automated pipelines and natural language processing technology into data collection routines, continuous efforts are made to reduce manual data entry burden and enhance quality control to ultimately increase efficiency and quality in data collection.

### A snapshot of contemporary arrhythmia therapy AF in a tertiary care center

This report of the baseline data and procedures performed in the first 1500 patients enrolled into TRUST provides a decent snapshot of contemporary arrhythmia management in a tertiary care center.

### AF ablation

Most patients (71%) underwent AF ablation, in line with the growing use of this intervention in Germany^22^ and Europe.^23,24^ A small majority underwent a first AF ablation after enrolment. The broad adoption of AF ablation reflects the growing confidence in pulmonary vein isolation as a safe and effective treatment strategy in patients with AF,^5,25^ but also highlights the continued need for repeat procedures after a first AF ablation.^26–30^ The age and comorbidity burden of this contemporary cohort of patients treated with AF ablation is higher than in most AF ablation trials (median age in TRUST 67 [58,75] years; median age on most ablation trials 60–66 years)^5,6,13,31–35^ except for CABANA, CAPLA, and the ablated patients in EAST-AFNET 4,^3,9,36^ but mirroring age ranges observed in other contemporary, large AF registries which commonly investigated effects of anticoagulation and therefore contain far less patients with prior catheter ablation (40% in TRUST, 2–7% in GARFIELD-AF and EORP-AF).^37,38^ These observations reflect the recent shift from traditional ablation patients, often young patients with structurally normal hearts apart from accessory pathways, abnormal AV nodes or idiopathic VES towards offering ablation to older patients with AF. This shift is also reflected by both a short time-frame between initial AF diagnosis until enrollment in TRUST (median 12 [4,41] months) and a comparably lower number of patients on antiarrhythmic drugs upon admission (23%) compared to prior and contemporary ablation trials.^6,13^ Recent clinical trials showed efficacy of catheter ablation in improvement of symptoms and reduction of adverse events in HFrEF patients.^4,33,36^ Ongoing outcome trials such as CABA-HFPEF-DZHK27^39^ and EASThigh-AFNET 11 will determine the safety and efficacy of AF ablation in patients with multiple comorbidities and AF. Cardiovascular risk factors and comorbidities were also broadly comparable: hypertension and diabetes prevalence, as well as coronary artery disease, prior myocardial infarction and rates of prior ischemic, embolic or bleeding events fell within the range reported across other large AF registries (see Supplementary material online, *Table S1*).^37,38,40–43^ However, heart failure prevalence was higher in TRUST compared to all other evaluated AF registries (≈60% in TRUST, 20–30% in other registries) which likely reflects more comprehensive phenotyping, particularly for HFpEF. As outlined, TRUST’s baseline assessment includes systematically performed echocardiography and HFpEF was defined by symptoms and echocardiography criteria as per respective 2021 ESC guidelines.^44^ Applying different diagnostic criteria for HFpEF on the TRUST data set revealed highly varying rates of HFpEF prevalence,^21^ however consistent with similar evaluations.^45,46^

### Supraventricular tachycardias

Supraventricular tachycardias remain a common reason for ablation procedures (16% of patients in TRUST). In TRUST, 69% of SVT patients underwent ablation within 1 month of inclusion, with a median age of 54 years and 46% females, also showing a shift towards older patients compared with contemporary^47^ and prior SVT cohorts since the broad adoption of SVT ablation in the 1990ies.^48,49^ Empirical slow pathway modulation, which was meanwhile adopted into SVT guidelines, is more routinely performed^49,50^ and also part of our strategy in TRUST patients when atrioventricular nodal reentry tachycardia was not inducible during the electrophysiological diagnostic procedure. Ongoing analyses will report outcomes of these patients.

### Ventricular arrhythmias

Ablation for PVC (95 patients) was more common than ablation for VT (35 patients), in line with recommendations to consider ablation as first-line therapy in idiopathic PVCs and as an important but later treatment option in patients with VTs.^51^ Patients undergoing PVC ablation were slightly younger (58 [48,70] vs. 59 [55,70]) and had fewer comorbidities than patients undergoing VT ablation (40/92 (43%) PVC procedures vs. 4/35 (11%) VT procedures with complete absence of structural heart disease), in line with previous reports in PVC^52,53^ and VT ablation.^54–57^ In TRUST, over two thirds of patients undergoing VT ablation suffered from heart failure, of which most had ischemic heart disease but one third suffered from dilatative cardiomyopathy. Over the half of these patients suffered from chronic kidney disease which was associated with worse short-term outcomes.^58,59^

### Medical therapy in ablation patients

Anticoagulation was commonly already initiated upon admission, especially in AF patients enrolled in TRUST. Anticoagulation was typically not interrupted peri-procedurally, and rate of patients on anticoagulation increased from admission to discharge across all sub-cohorts. Antiarrhythmic drug therapy was often continued. The observed discontinuation rates are slightly higher than those reported by others,^60,61^ but these are balanced by reinitiation of new AADs. Patients with structurally normal hearts undergoing VT or PVC ablation rarely received AAD therapy prior to enrolment, in line with recommendations.^51^

### Long-term follow-up

Participants will be followed up for longer periods, providing information on disease progression and patient well-being after initial therapy. Clinical trials, exemplarily in the field of interventional treatment of AF, are limited in their follow-up durations of one^25,35^ to less than five years^5,36,62,63^ as appropriate for their respective research questions. Treatment of AF patients at specialized centers often only starts many months after disease diagnosis, as shown by a long median time from first diagnosis to TRUST inclusion (35 months in this snapshot analysis). One exemplary field of complexity that remains challenging is the ongoing treatment of patients with AF after multiple invasive procedures.^29^ Longer-term follow-up in TRUST will provide valuable information on patient trajectories after a first and repeat AF ablation, including temporal trends and treatment patterns. As guidelines are being updated during the course of TRUST,^4,64,65^ its design will also enable analysis of adoption and validation of efficacy and safety of new guideline recommendations in real-world scenarios.

### Embedded routine biobanking

TRUST’s biobank is another key strength, with baseline biobanking available in 72% of the first 3002 participants. Biomarkers have great potential as quantifiable proxies of disease processes with conceptually near-universal availability^66–70^ and can be linked to prevalent diseases^66^ and outcomes.^67^ Linking clinical characteristics, imaging, and electrophysiological data from TRUST with blood-based biomarkers offers opportunities to generate pathophysiological hypotheses. Analyses of blood biomarkers in selected patients enrolled in TRUST already enabled differentiation of acute and chronic myocardial injury^71^ and quantification of myocardial damage after AF ablation using different ablation systems.^72^ Further analyses of these and other blood biomarkers will be conducted in TRUST.

Through the adoption of novel technologies, particularly remote monitoring tools, TRUST captures valuable continuous data from patients outside hospital settings. This approach not only improves the efficiency of tracking arrhythmia recurrences but also provides an effective method for characterizing disease burden and progression after treatment,^12,73,74^ and enables another method for ascertaining living status and collecting information on events during follow-up.

## Strengths and limitations

A key strength of TRUST is the homogeneous, deep phenotyping at baseline, standardized procedures for the collection of biosamples, and availability of digital ECG and imaging data, combined with blended follow-up combining opportunistic in-person visits with online follow-up for events and quality of life. A major limitation is the single-center setting in a tertiary care university medical center with low-threshold access to interventional treatment of arrhythmias. This leads to a high proportion of patients treated with ablation procedures in the cohort. Other data sets generated in multiple and diverse centers and electronic health record data sets provide more generalizable findings,^22,75–77^ albeit with less detailed phenotype information. Further, the large number of enrolled patients with different types of cardiac arrhythmias exposes a significant workload, especially as TRUST is investigator-initiated and receives no specific funding for its infrastructure and maintenance, but certain externally funded sub-projects based on data collection in TRUST contribute to this. The high proportion of patients that can only undergo remote follow-up, with only a selection of patients that can be followed up via inpatient visits, as well as the intermittent pausing of recruitment during follow-up, reflects this lack of resources. Because urgent, non-elective cases could sometimes not be approached timely manner, some selection toward more stable elective patients is possible. However, this potential bias arises only from workflow constraints inherent to an embedded clinical registry driven by clinicians rather than systematic exclusion of specific patient groups. The broad enrolment and harmonized data collection enables analyses of disease specific and shared follow-up pathways, study of rarer arrhythmias, and analyses across patients with different arrhythmias to study their combined prevalence and possible interactions.^78–80^ The integration of TRUST with other cohort studies at the University Heart and Vascular Center Hamburg, including shared infrastructure, data, and biosamples, and comparative analyses across cardiovascular cohorts and diseases are feasible.^81,82^

## Conclusion

The TRUST study combines detailed, homogeneous phenotyping of consecutive patients with arrhythmias with long-term outcome tracking using blended methods in a real-world setting. Leveraging advanced diagnostics and data analysis technologies, including machine learning and deep learning, this rich data set can enhance our understanding of arrhythmia management. The high rate of participant enrollment and biobanking indicates strong engagement and the potential for substantial contributions to both clinical practice and academic research, especially in view of the integration with other disease cohorts at the UHZ.

## Lead author biography


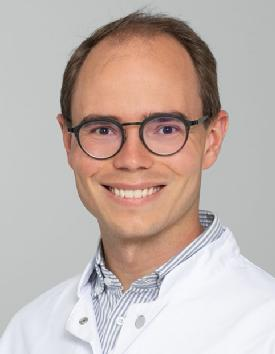


Dr. Julius Obergassel, MD, MHBA, is a cardiology fellow and clinician scientist at the University Heart and Vascular Center of the University Medical Center Hamburg-Eppendorf. His clinical and research focus is on atrial fibrillation, electrophysiology, cardiovascular data science and real-world evidence generation. He co-leads a cross-disciplinary lab developing mathematical and artificial intelligence approaches for biomarker discovery and clinical decision support. He is actively involved in clinical trials and outcomes research in arrhythmia care and has expertise in their design, governance, and implementation. In 2024, he co-founded IDM gGmbH, a non-profit subsidiary of the University Medical Center Hamburg-Eppendorf, developing AI solutions for use in routine healthcare.

## Supplementary Material

oeag002_Supplementary_Data

## Data Availability

The full data underlying this article cannot be shared publicly due to the privacy of individuals that participated in the study. Parts of data, when de-identifiable, will be shared on reasonable request to the corresponding author.
