# Supplementary material for: Long-term outcome and predictors for recurrence after medical and interventional treatment of arrhythmias at the UniverSity Heart CenTer Hamburg (TRUST): design and patient profile snapshot of a prospective clinical cohort study
Source: Eur Heart J Open. 2026 Jan 14;6(1):oeag002. doi: 10.1093/ehjopen/oeag002 (PMC12930198; doi:10.1093/ehjopen/oeag002)
Supplement: oeag002_Supplementary_Data [file oeag002_supplementary_data.docx]

Long-**T**erm Outcome and Predictors for **R**ecurrence after Medical and Interventional treatment of Arrhythmias at the **U**niver**S**ity Heart Cen**T**er Hamburg (TRUST): Design and patient profile snapshot of a prospective clinical cohort study

Running title: Design and patient profile snapshot of TRUST

Julius Obergassel, MD ^1,2^, Jan Leon Rieß, MD ^1,2^, Sandro Jaeckle ^1^, Silke van Elferen ^1,2,3^, Moritz Nies, MD ^1,2^, Niklas Schenker, MD ^1,2^, Marc Daniel Lemoine, MD ^1,2^, Alexander Welcker, MD ^1,2^, Shinwan Kany, MD ^1,2^, Laura Rottner, MD ^4^, Djemail Ismaili, MD ^1,2^, Laura Charlotte Sommerfeld, PhD ^1,2^, Johannes Petersen, MD ^5,2^, Katharina Govorov ^1^, Lauritz Schoof, MD ^1^, Olga Tsoy ^3^, Jan Baumbach ^3,6^, Simon Pecha, MD ^1,2^, Tanja Zeller, MD ^2,7^, Stefan Blankenberg, MD ^1,2^, Larissa Fabritz, MD ^1,2,8^, Bruno Reißmann, MD ^1,2^, Feifan Ouyang, MD ^1^, Andreas Rillig, MD ^1,2^, Andreas Metzner, MD ^1^, Paulus Kirchhof, MD ^1,2,8^

1. Department of Cardiology, University Heart & Vascular Center Hamburg, University Medical Center Hamburg-Eppendorf, Hamburg, Germany
2. German Center for Cardiovascular Research (DZHK), partner site Hamburg/Kiel/Lübeck, Germany
3. Institute for Computational Systems Biology, Universität Hamburg, Hamburg, Germany
4. Department of Cardiology, University Hospital Frankfurt, Frankfurt, Germany
5. Department of Cardiovascular Surgery, University Heart & Vascular Center Hamburg, University Medical Center Hamburg-Eppendorf, Hamburg, Germany
6. Computational Biomedicine Lab, Department of Mathematics and Computer Science, University of Southern Denmark, Odense, Denmark
7. Institute for Cardiogenetics, Institute for Genetics, University Hospital Schleswig-Holstein, Campus Lübeck, Lübeck, Germany
8. Institute of Cardiovascular Sciences, University of Birmingham, United Kingdom

# Supplemental Material

**Address for correspondence**

Dr. Julius Obergassel, M.D., MHBA

University Medical Center Hamburg-Eppendorf

University Heart & Vascular Center Hamburg

Department of Cardiology

Martinistr. 52, 20246 Hamburg, Germany

Phone: +49 (0) 40 – 7410 0

E-Mail: j.obergassel@uke.de

## Supplemental Tables

**Supplemental Table 1: Comparison of baseline characteristics between the atrial fibrillation (AF) sub-cohort within the TRUST registry and contemporary AF registries GARFIELD-AF**^1^**, GLORIA-AF**^2^**, PREFER in AF**^3^**, EORP-AF**^4^**, FUSHIMI-AF**^5^ **and ORBIT-AF**^6^**.**

|  | **TRUST**  **AF and AT** | **GARFIELD-AF** | **GLORIA-AF**  **(phase 3)** | **PREFER in AF** | **EORP-AF** | **FUSHIMI-AF**  **(2013)** | **ORBIT-AF** |
| --- | --- | --- | --- | --- | --- | --- | --- |
| **N participants (%)** | 1,077 (72) | 52,032 (100) | 21,241 (100) | 7,243 (100) | 11,096 (100) | 3,183 (100) | 10,135 (100) |
| **Age** | 67 [58,75] | 71 [63,78] | 71 ± 11 | 71.5 ± 11 | 71 [63,77] | 74 ± 11 | ♀77[69,83] ♂73[65,80] |
| **Female sex, N (%)** | 385 (36) | 22,989 (44) | 9,546 (44.9) | 39.8% | 4,512 (41) | 1,296 (41) | 4,293 (42) |
| **Body mass index (kg/m^2^)** | 27 [24,31] | 26.9 [24,31] | 29 ± 6 | n/a | n/a | 23 ± 4 | ♀28[24,34] ♂29[26,34] |
| **CHA_2_DS_2_-VA*(Sc)* Score, median and/or mean** | 3 [2,4]  2 (2) | *3 [2,4]* | n/a | *3 (2)* | *3 [2,4]* | *2 ± 4* ^†^ | *♀ 5 [4,6]*  *♂ 3 [2,5]* |
| **Heart rhythm** | | | | | | | |
| **Atrial fibrillation, N (%)** | 1,077 (100) | 52,032 (100) | 21,241 (100) | 7243 (100) | 11,096 (100) | 3,183 (100) | 10,135 (100) |
| **Paroxysmal atrial fibrillation, N (%)** | 424 (40) | 14,307 (28) | 11,972 (56) | 30% | 2,850 (26) | 1,463 (46) | 5,119 (51) |
| **Prior catheter ablation, N (%)** | 435 (29) | n/a | 382 (2) | 358 (5%) ^††^ | 490 / 10,577 (5) | 168 (5) | 552 (5) |
| **Present Heart Disease** | | | | | | | |
| **Heart failure, N (%)** | 725 (67) ^§^ | 11,739 (23) ^§§^ | 4,616 (22) ^§§§^ | 21% ^§§§^ | 4,343 (40) ^§§§^ | 889 (28%) ^§§§§^ | 3,288 (32) ^§§§^ |
| **Coronary artery disease, N (%)** | 286 (26) | 11,253 (22) | 3,967 (19) | 23% | 3,058 (29) | 479 (15) | 3,645 (36) |
| **Prior myocardial infarction, N (%)** | 84 (8) | 5,536 (11) | n/a | 11% | 1,347 (12) | 203 (6) | n/a |
| **Valvular heart disease, N (%)** | 256 (24) | n/a | n/a | n/a | 5,527 (51) ^†††^ | 576 (18) | 832 (8) ^††††^ |
| **Medical History** | | | | | | | |
| **Arterial hypertension, N (%)** | 751 (70) | 39,610 (76) | 15,515 (73) | 72% | 6,831 (62) | 1,928 (61) | 8,415 (83) |
| **Diabetes mellitus, N (%)** | 125 (12) | 11,546 (22) | 4,940 (23) | 23% | 2,537 (23) | 737 (23) | 2,983 (29) |
| **Hypercholesterinemia, N (%)** | 449 (42) | 20,959 (42) | 8,296 (39) | n/a | 4,392 (41) | 1,350 (42) | 7,285 (72) |
| **Peripheral artery disease, N (%)** | 37 (3.4) | n/a | n/a | 4.4% | 883 (8) | 138 (4) | n/a |
| **Pulmonary embolism, N (%)** | 33 (3.1) | 1,354 (3) ^#^ | n/a | n/a | n/a | n/a | n/a |
| **Chronic kidney disease, N (%)** | 300 (28) ^##^ | 5,355 (12) ^###^ | 389 (2) ^####^ | 10% ^##^ | 1,379 (13) | 841 (26) ^#####^ | n/a |
| **Prior major bleeding (ISTH or equivalent), N (%)** | 29 (3) | 1,316 (3) | n/a | 7.3% | 572 (5) | 55 (2) | 572 (5) |
| **Prior stroke or TIA, N (%)** | 93 (9) | 6,145 (12) | 3,086 (15) | 16% | 1,012 (9) | 619 (19)/ 75 (2) | 1,531 (15) |
| **Any malignant disease, N (%)** | 155 (14) | n/a | 2,112 (10) | n/a | 818 (7) | n/a | n/a |
| **Hyperthyroidism, N (%)** | 47 (4) | 898 (2) | n/a | n/a | 497 (5) | n/a | n/a |
| **Hypothyroidism, N (%)** | 39 (4) | 3,035 (6) | n/a | n/a | 1,031 (10) | n/a | n/a |
| † = CHADS_2_ score, excluding ; †† = Limited to past 12 months; ††† = “Valvular alterations”, included low-grade disease; †††† = Prior valve repair or replacement; # = Includes deep vein thrombosis; ## = Estimated glomerular filtration rate < 60 ml/min/1.73m^2^ or physician-reported chronic kidney disease; ### = Limited to moderate or severe chronic kidney disease; #### Chronic dialysis or renal transplantation or serum creatinine ≥ 200 μmol/L; ##### = perisitent proteinuria or glomerular filtration rate < 60 ml/min/1.73m^2^ for more than 3 months, | | | | | | | |
| **Heart failure (HF) definitions in different registries**  § = Left-ventricular ejection fraction < 50% *or* left-ventricular ejection fraction ≥ 50% but at least one symptom/sign of HF with preserved ejection fraction (HFpEF) + at least one cardiac function criterium according to European Society of Cardiology’s definition of HFpEF *(rhythm-dependent N-terminal pro-natriuretic peptide cut-offs; body-surface-indexed left atrial volume; early filling velocity on transmitral doppler divided by early relaxation velocity on tissue doppler at rest; body-surface-indexed left ventricular mass; relative wall thickness; systolic pulmonary artery pressure or – only if other criteria not available –* physician-reported HF and HF class  §§ = Current or prior heart failure *or* left-ventricular ejection fraction < 40%  §§§ = Physician-reported heart failure  §§§§ = Prior heart failure hospitalizations *or* heart failure symptoms *or* left-ventricular ejection fraction < 40% | | | | | | | |

## Text Supplement 1

### Echocardiography protocol (baseline and follow-ups)

Transthoracic echocardiography is routinely performed upon inclusion by trained cardiologists and includes acquisition of all standard views, including a complete 3-dimensional-volume over four beats, focused views on the left and right atrium with and without tissue doppler imaging. Left and right ventricular, as well as left and right atrial strains are assessed as previously described ^7^. Identified pathologies are further and specifically investigated. All TRUST-related echocardiography studies are stored pseudonymized in their original acquisition format for additional further analyses, including machine learning based analyses.

### Biobanking protocol (baseline and follow-ups)

Biosamples collected in TRUST mainly include blood, as well as waste tissue from cardiac surgeries. Biosamples are collected upon inclusion in all participants. Additional serum samples (15 mL) are collected in selected patients at the start and the end of ablation procedures. Participants returning for in-house follow-ups receive an additional blood draw at the respective follow-up. Blood is centrifuged at 3000 rpm for 10 minutes, aliquoted and stored in the long-term freezers at -80 °C within 4 hours after sampling. All biosamples collected for TRUST will be stored for up to 60 years.

Waste tissue of the left atrium (LA) and / or its appendage is collected from participants included in the cardiac surgery sub-cohort of TRUST. Tissue is collected from the surgery hall by trained study personnel, immediately shock-frozen in liquid nitrogen and then stored at -80 °C. The sampling and storage procedures will allow analyses for a wide range of blood biomarkers ^8,9^.

### Neurocognitive assessment (baseline and follow-ups)

The Montreal Cognitive Assessment (MoCA) test has been evaluated and established as a screening instrument for the detection of cognitive impairment ^10–12^. Baseline neurocognitive function of TRUST participants is assessed at the day of inclusion by trained study personnel, as intended ^12^. Follow-up assessment of neurocognitive status is performed remotely via MiniMoCA ^13^, as well as via the original MoCA test during in-house follow-up visits.

### Questionnaires (baseline and follow-ups)

Questionnaires are filled by all participants regarding further demographics, personal and family’s medical history, personal and familiar cardiovascular and neurocognitive risk factors, personal quality of life (QoL), depressive symptoms, more detailed questions regarding their clinical arrhythmia-related symptoms. QoL assessment is performed via the Atrial Fibrillation Effect on QualiTy-of-life (AFEQT) questionnaire ^14^, as well as the EQ-5D-5L ^15^ for the QoL in participants with HF-symptoms. Symptoms of depression is tested upon inclusion using the PHQ-9 questionnaire ^16^. QoL assessment is repeated at all remote and in-house follow-ups.

All self-administered questionnaires are provided via tablets connected to the internal network to participants, as well as in written form upon request, again ensuring high data quality via the reduction of manual data entry. Questionnaires are provided using the survey functionality of the REDCap electronic data capture platform^17^, enhanced by custom-developed tools for the integration in the hospital workflow and to ensure pseudonymization.

## Text Supplement 2: Automated and Semi-Automated Data Processing and AI-Supported Feature Extraction

To support standardized data collection within TRUST, several automated and semi-automated workflows are applied within the protected research environment.

Pseudonymized clinical data and patient-reported or -collected information are processed using an in-house tool that manages hierarchical pseudonymization, organizes study-related workflows for notifications of planned or unplanned re-hospitalizations, and enables structured incorporation of wearable- and smartphone-derived data.

Digital ECGs are routinely available, and manufacturer-provided feature sets are automatically integrated in our database. In selected sub-projects, additional ECG-based features and interpretations are generated using locally deployed, open-weight deep learning models. Certain available, unstructured clinical text, including procedural and diagnostic reports, is processed using natural-language-processing pipelines based on regular expressions, stochastic natural-language-processing techniques and locally hosted language models. These pipelines extract structured variables and perform basic classification tasks. All automatic processing pipelines are deployed within the institution’s secure research infrastructure, currently without any use of external or cloud-based artificial intelligence providers or services.

Currently, these models and pipelines are currently evaluated in project-level implementations and only in human-in-the-loop workflows to reduce manual data entry requirements and enable reproducible, privacy-preserving enrichment of TRUST’s structured research dataset systematically and across the full cohort in the future.

## Supplemental References

1. Fox, K. A. A. *et al.* GARFIELD-AF risk score for mortality, stroke, and bleeding within 2 years in patients with atrial fibrillation. *European Heart Journal - Quality of Care and Clinical Outcomes* **8**, 214–227 (2022).

2. Beier, L. *et al.* Evolution of antithrombotic therapy for patients with atrial fibrillation: The prospective global GLORIA-AF registry program. *PLoS ONE* **17**, e0274237 (2022).

3. Kirchhof, P. *et al.* Management of atrial fibrillation in seven European countries after the publication of the 2010 ESC Guidelines on atrial fibrillation: primary results of the PREvention oF thromboemolic events—European Registry in Atrial Fibrillation (PREFER in AF). *EP Europace* **16**, 6–14 (2014).

4. Boriani, G. *et al.* Contemporary stroke prevention strategies in 11 096 European patients with atrial fibrillation: a report from the EURObservational Research Programme on Atrial Fibrillation (EORP-AF) Long-Term General Registry. *EP Europace* **20**, 747–757 (2018).

5. Akao, M. *et al.* Current status of clinical background of patients with atrial fibrillation in a community-based survey: The Fushimi AF Registry. *Journal of Cardiology* **61**, 260–266 (2013).

6. Piccini, J. P. *et al.* Differences in Clinical and Functional Outcomes of Atrial Fibrillation in Women and Men: Two-Year Results From the ORBIT-AF Registry. *JAMA Cardiol* **1**, 282 (2016).

7. Knappe, D. *et al.* Association of atrial mechanical dispersion with atrial fibrillation recurrence following catheter ablation: results of the ASTRA-AF pilot study. *Clin Res Cardiol* (2024) doi:10.1007/s00392-024-02435-0.

8. Chua, W. *et al.* An angiopoietin 2, FGF23, and BMP10 biomarker signature differentiates atrial fibrillation from other concomitant cardiovascular conditions. *Sci Rep* **13**, 16743 (2023).

9. Fabritz, L. *et al.* Biomarker-based prediction of sinus rhythm in atrial fibrillation patients: the EAST-AFNET 4 biomolecule study. *European Heart Journal* **45**, 5002–5019 (2024).

10. Bailey, M. J. *et al.* Relation of Atrial Fibrillation to Cognitive Decline (from the REasons for Geographic and Racial Differences in Stroke [REGARDS] Study). *The American Journal of Cardiology* **148**, 60–68 (2021).

11. Ciesielska, N. *et al.* Is the Montreal Cognitive Assessment (MoCA) test better suited than the Mini-Mental State Examination (MMSE) in mild cognitive impairment (MCI) detection among people aged over 60? Meta-analysis. *Psychiatr Pol* **50**, 1039–1052 (2016).

12. Nasreddine, Z. S. *et al.* The Montreal Cognitive Assessment, MoCA: A Brief Screening Tool For Mild Cognitive Impairment. *J American Geriatrics Society* **53**, 695–699 (2005).

13. Campbell, N., Rice, D., Friedman, L., Speechley, M. & Teasell, R. W. Screening and facilitating further assessment for cognitive impairment after stroke: application of a shortened Montreal Cognitive Assessment (miniMoCA). *Disability and Rehabilitation* **38**, 601–604 (2016).

14. Spertus, J. *et al.* Development and validation of the Atrial Fibrillation Effect on QualiTy-of-Life (AFEQT) Questionnaire in patients with atrial fibrillation. *Circ Arrhythm Electrophysiol* **4**, 15–25 (2011).

15. Stolk, E., Ludwig, K., Rand, K., Van Hout, B. & Ramos-Goñi, J. M. Overview, Update, and Lessons Learned From the International EQ-5D-5L Valuation Work: Version 2 of the EQ-5D-5L Valuation Protocol. *Value in Health* **22**, 23–30 (2019).

16. Kroenke, K., Spitzer, R. L. & Williams, J. B. W. The PHQ-9: Validity of a brief depression severity measure. *J Gen Intern Med* **16**, 606–613 (2001).

17. Harris, P. A. *et al.* The REDCap consortium: Building an international community of software platform partners. *Journal of Biomedical Informatics* **95**, 103208 (2019).
